# Methods and approaches in the topology-based analysis of biological pathways

**DOI:** 10.3389/fphys.2013.00278

**Published:** 2013-10-10

**Authors:** Cristina Mitrea, Zeinab Taghavi, Behzad Bokanizad, Samer Hanoudi, Rebecca Tagett, Michele Donato, Călin Voichiţa, Sorin Drăghici

**Affiliations:** ^1^Department of Computer Science, Wayne State UniversityDetroit, MI, USA; ^2^Department of Obstetrics and Gynecology, Wayne State UniversityDetroit, MI, USA

**Keywords:** pathway analysis, topology, signaling pathways, metabolic pathways, mathematical model, network topology, statistical significance

## Abstract

The goal of pathway analysis is to identify the pathways significantly impacted in a given phenotype. Many current methods are based on algorithms that consider pathways as simple gene lists, dramatically under-utilizing the knowledge that such pathways are meant to capture. During the past few years, a plethora of methods claiming to incorporate various aspects of the pathway topology have been proposed. These topology-based methods, sometimes referred to as “third generation,” have the potential to better model the phenomena described by pathways. Although there is now a large variety of approaches used for this purpose, no review is currently available to offer guidance for potential users and developers. This review covers 22 such topology-based pathway analysis methods published in the last decade. We compare these methods based on: type of pathways analyzed (e.g., signaling or metabolic), input (subset of genes, all genes, fold changes, gene *p*-values, etc.), mathematical models, pathway scoring approaches, output (one or more pathway scores, *p*-values, etc.) and implementation (web-based, standalone, etc.). We identify and discuss challenges, arising both in methodology and in pathway representation, including inconsistent terminology, different data formats, lack of meaningful benchmarks, and the lack of tissue and condition specificity.

## 1. Introduction

In molecular biology and genetics, there is a large gap between current data analysis techniques and their ability to derive precise and accurate functional information from the large and constantly growing volume of high throughput molecular data. The capability of obtaining a comprehensive lists of genes/proteins that are different between two phenotypes is routine^1^ in research today. And yet, the holy grail of high-throughput has not delivered so far. Even though high-throughput comparisons are relatively easy to perform, understanding the phenomena that determine the measured changes is as challenging as ever, if not more so. Therefore, it is crucial to develop effective ways to analyze the vast amount of data that has been and will continue to be collected.

A major contributor to the gap between our ability to collect data and our ability to interpret it, is the fact that living organisms are complex systems whose emerging phenotypes are the results of thousands of complex interactions taking place on various metabolic and signaling pathways. The ability to correctly infer the perturbed pathways responsible for a phenotype from a list of differentially expressed (DE) genes or proteins may be the key to transforming the now abundant high-throughput expression data into biological knowledge. In turn, this can help understand mechanisms of disease, develop better drugs, personalize drug regimens, etc. For our purposes, pathways are models describing the interactions of genes, proteins, or metabolites within cells, tissues, or organisms, not simple lists of genes. This is why, in this paper, we focus exclusively on pathway analysis methods that aim to identify the pathways that are significantly impacted in a condition under study, taking pathway topology into account. This process uses two types of data: (i) previously accumulated knowledge in the form of known pathways, represented as graphs and (ii) experiment data, such as gene expression values or protein or metabolite abundance data obtained when comparing two phenotypes.

In spite of the crucial importance of this problem and of the recent increase in the number of methods and approaches for pathway analysis, to our knowledge there is no current review focused on topology-based methods. A reason for this may be related to the challenges currently associated with this problem. A first such challenge is the lack of standards for the evaluation of the results of the analyses. This has lead to the proliferation of many techniques that have never been compared with each other in a consistent way. Another set of challenges is related to the pathways themselves. Not only there is no universal agreement for the representation of information content in pathway databases, but the very definition of a pathway is not completely agreed upon (Chowbina et al., 2009). Some authors use the term “pathway” to refer to a simple list of genes (such as those associated with a given Gene Ontology (GO) term), lacking any structure and any information about the interactions between these genes. Many others use graphs to capture relationships but the meaning of edges and nodes varies dramatically from one source to another. Figure 5 shows not fewer than five different types of graphs, all referred to as “pathways.” Even pathways from the same source, often use different representations. For instance, genes/proteins are associated with nodes in KEGG signaling pathways while they are associated with edges in KEGG metabolic pathways.

**Figure 1:**
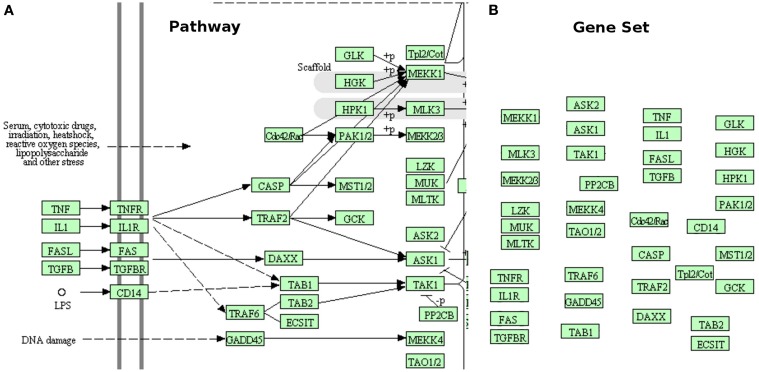
**Gene sets are not pathways. (A)** shows a small part of the MAPK signaling pathway from KEGG. This pathway shows the location of various genes or gene products (inside the cell, outside of it, or in the membrane), what gene interacts with what other gene(s), the type of each interaction (activation, repression, phosphorylation, etc.), the direction of the signal propagation, and potentially many other things (e.g., complex formation, etc.). **(B)** presents the same part of the same pathway as a gene set (no interactions). The gene set has lost all the structure and the additional information captured by the original pathway. This comparison shows how much important knowledge existent in pathway database is ignored when pathways are treated as simple gene sets.

The subset of available techniques that consider the pathways as simple lists of genes, such as those associated with a GO term (or another arbitrary descriptor) are worth of further discussion. Here, we will refer to these as gene set analysis methods, rather than pathway analysis methods. A comprehensive list of such techniques, as well as some comparisons between them can be found in several well-developed surveys (Misman et al., 2009; Chuang et al., 2010; Kelder et al., 2010; Emmert-Streib and Glazko, 2011; Khatri et al., 2012). While useful for the purpose for which they have been developed - to analyze sets of genes - these methods do not take into consideration the topology of the pathways, and hence completely ignore the interactions described by the pathways, the different types of genes, the position of the genes on their respective pathways, etc. This is illustrated in Figure 1. In some sense, the very reason for the existence of the pathways is to describe the way various genes interact. Therefore, methods that perform the analysis only on sets of genes, ignoring the topology of the pathway, are not included in the scope of the present review.

**Figure 2:**
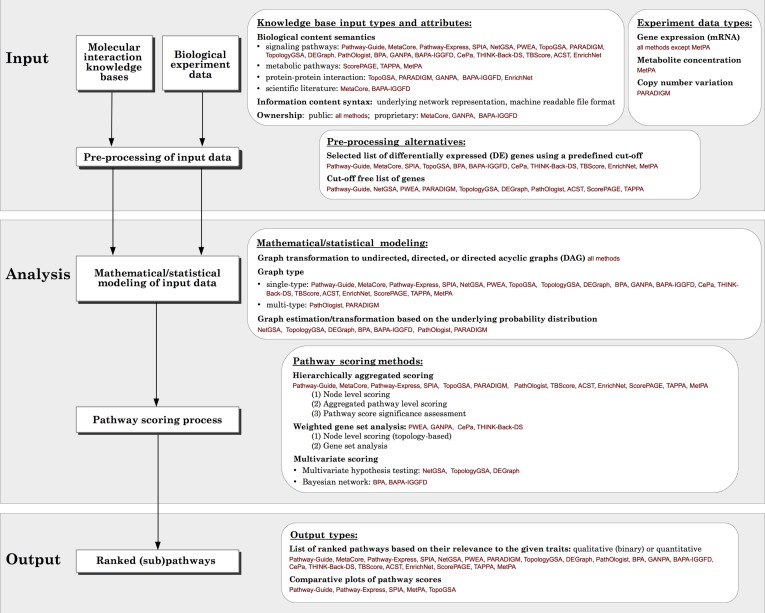
**Generalized overview of the data flow in pathway analysis methods.** For each module, the various options available for different methods surveyed, as well as the comparison criteria used in this paper are presented in the white boxes.

Recent pathway analysis algorithms have become more refined than gene set analysis methods by incorporating topology (Figure 2). A first attempt to incorporate topology information in the analysis of pathways was through the use of graph theory methods. This approach became popular in the last decade (Chuang et al., 2010; Barabási et al., 2011). Aittokallio and others survey graph-based analysis methods. They identify categories based on global structural properties, local structural connectivity, or hierarchical functional organization, and describe the features of gene regulatory networks, metabolic networks, and protein-protein interaction (PPI) networks (Aittokallio and Schwikowski, 2006). Some of these graph theory methods and concepts are relevant to the pathway analysis methods able to compare phenotypes, which are the focus of the current review. However, as a broad category, the approaches based on graph theory methods are not able to identify the pathways that are significant in a given phenotype comparison and therefore, do not fall within the scope of this review.

Varadan and others (Varadan et al., 2012) review the use of biological knowledge bases for cancer diagnosis and prognosis. They attempt to evaluate the performance of three topology-based methods, SPIA, PARADIGM, and PathOlogist, on the same input datasets to compare the biological relevance of their outputs. Unfortunately, since the 3 tools did not use the same pathway database, the authors chose to re-implement SPIA and adapt it to the pathway database used by the other two, so that the result from all three would be comparable. The authors discuss relative performance of the three methods, but could not draw definitive conclusions regarding the superiority of one tool versus another. We also ran their version of SPIA and the original SPIA implementation from Bioconductor, using exactly the same input, and obtained different results. This indeed demonstrates some of the inherent problems encountered when comparing pathway analysis methods. First of all, it is difficult to successfully re-implement an algorithm to force it to work on other data sources, especially when the re-implementation is done by third parties. Furthermore, sometimes the mere ability to reproduce published results - which is at the base of modern scientific research - is questionable in this area. For instance, in spite of having access to the source code and having the full cooperation of the authors, we could not even reproduce the results reported in Vaske et al. (2010).

Four topology-based tools, along with several gene-based methods, were recently reviewed by Khatri and others Khatri et al. (2012). This recent survey groups functional analysis based on GO together with pathway analysis methods. With this very loose definition of a pathway and pathway analysis, the authors present the limitations and challenges of various methods in general, and categorize topology-based methods as “third-generation” tools. However, even though it is very recent, this existing survey only includes 4 out of the 22 topology-based analysis methods reviewed here.

In a different direction, researchers tackle the problem of understanding disease by looking at signaling networks from the perspective of fault tolerance. Fault tolerance is a measure of the vulnerability of signaling networks to the abnormal function of its components. Abdi and Emamian survey this direction in a comprehensive study Abdi and Emamian (2010). Valuable results are presented highlighting vulnerable molecules in different molecular networks for biological phenomena such as mitosis or p53 signaling.

Kinetic/stoichiometric models based on the molecular mechanisms of interaction have been used for over 25 years in order to simulate biochemical phenomena. Such models are in some sense the ultimate tools because they can predict exact quantities for any variable in the system. However, their use is limited by the need to know the precise initial concentration for most reactants, exact reaction constants for all reactions, as well as the appropriate time scale for the studied phenomenon. Furthermore, the goals of such models are very different from the goals of pathway analysis methods. The goal of such kinetic models is to fully describe the biochemical phenomena involved and to make quantitative predictions about some of the reaction products involved. In contrast, the goal of pathway analysis methods is to identify the most significantly impacted pathways from a large collection of heterogeneous pathways, based on incomplete information. Furthermore, kinetic models work for biochemical pathways describing reactions of the same type (biochemical) with known reaction constants (Steuer, 2007). The pathways we are considering here include gene signaling pathways containing different “signals” (inhibition, activation, phosphorylation, methylation, etc.) happening at many levels (transcription, translation, post-translational, etc.) between heterogeneous components (mRNA, DNA, protein, metabolites, etc.). Therefore, the entire body of work concerned with modeling biochemical pathways using mathematical models (e.g., differential or difference equations) does not fall within the scope of this review.

Finally, it is important to state that we do not intend to assess the efficacy of each method, since there is not a universally recognized correct output of such tools. Designing benchmark datasets would help to determine the most effective mathematical model but this is beyond the intended scope of the current review and hence, it is not attempted here.

**Figure 3:**
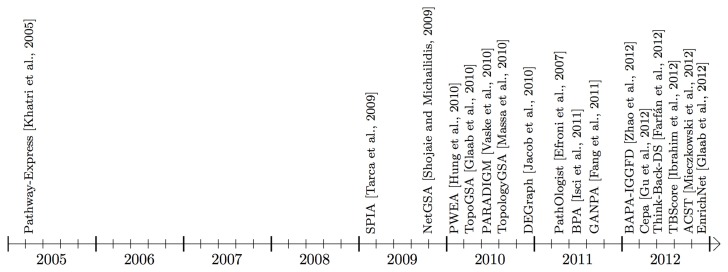
**Timeline showing when the surveyed pathway analysis tools, working mainly with signaling pathways, became available (this time may be different from publication time shown in Table 1).** Some of the methods use additional interaction information that may be from an in-house or public gene/protein interaction knowledge base. BAPA-IGGFD (Zhao et al., 2012) and TBScore (Ibrahim et al., 2012) acronyms were assigned to the respective methods, in this manuscript, for ease of reference. The commercial tools, Pathway-Guide and MetaCore are not included in this figure.

**Figure 4:**
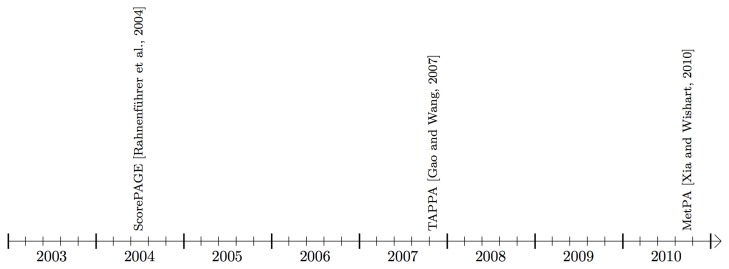
**Timeline showing the availability of pathway analysis tools that work mainly with metabolic pathways**.

In this paper, we describe 22 topology-based pathway analysis methods designed to analyze either signaling pathways (see Figure 3), or metabolic pathways (Figure 4). There are several commercial tools used for pathway analysis, which do not incorporate the pathway topology when computing pathway scores including Ingenuity Pathway Analysis (Ingenuity Systems, www.ingenuity.com) and Genomatix (Genomatix Software, www.genomatix.de). Since these tools only perform a gene set analysis, failing to take advantage of the additional knowledge incorporated in the pathways, they will not be considered here. We found only two commercial tools that do incorporate topology in the pathway analysis. These are Pathway-Guide (Advaita Corporation, http://www.advaitabio.com) and MetaCore (Thomson Reuters, http://www.thomsonreuters.com).

We categorize and compare all surveyed methods based on different criteria including: the type of input required, the type of output provided, the mathematical models used, and the implementation used. In section 2, we discuss the options for input data in different tools, in particular, the challenges specific to topology-based methods. Section 3 reviews the underlying mathematical models and scoring methods currently available to rate pathway deregulation. Section 4 focuses on the types of output provided. Finally, section 5 presents issues regarding the implementation of the methods. To the best of our knowledge, our review is the only comprehensive survey of topology-based pathway analysis methods to date.

## 2. Input data

This review focuses on pathway analysis methods that try to exploit some of the information contained in the pathway topology in order to identify the pathways that are significantly impacted in a condition under study. In order to address this problem, any pathway analysis method will need: (i) a collection of pathways capturing our current knowledge about the interactions of genes, proteins, metabolites, or compounds in an organism (usually from a pathway database), and (ii) experimental data in the form of measurements of gene expression, protein abundance, metabolite concentration, or copy numbers. The pathway data is accumulated, updated, and refined by amassing knowledge from scientific literature describing individual interactions or high throughput experiment results. The experiment data is usually provided by measurements comparing two or more phenotypes such as treated vs. untreated, disease vs. healthy, or treated with drug A vs. drug B.

Analysis methods take various approaches to accommodate the different formats commonly used for both types of data. In this section, we compare all methods reviewed based on their input types and formats, and discuss the particular difficulties encountered when incorporating the pathway interactions into topology-based analysis methods.

### 2.1. Experiment data

Most methods analyze data from high-throughput experiments, such as microarrays, next-generation sequencing, or proteomics. Most analysis methods accept either a list of gene IDs or a list of such gene IDs associated with measured changes. These changes could be measured with different technologies and therefore can serve as proxies for different biochemical entities. For instance, one could use gene expression changes measured with microarrays, or protein levels measured with a proteomic approach, etc. Transcription data is often used to approximate the proteome, since high-throughput protein abundance data is not readily available. Most methods expect a consistent input i.e., all values are expected to be of the same type. MetPA, which is a metabolic pathway analysis method, is the only method that does not accept gene expression. This method uses as input either a list of “important” compounds, or a metabolite concentration table.

Different analysis methods use different input formats. Many methods accept a list of all genes considered in the experiment together with their expression values. Some analysis methods select a subset of genes, considered to be differentially expressed (DE), based on a predefined cut-off. The cut-off is typically applied on fold-change, statistical significance, or both. A selection based on both criteria can be performed easily if the data is displayed as a volcano plot, i.e., in a coordinate system that has fold changes on the x axis and the negative log of the *p*-value on the y axis. In such a plot, genes that have large absolute fold changes as well as significant *p*-values will appear in the top part of the plot, towards the sides. These methods use the list of DE genes and their corresponding fold-change values as input. Other methods use only the list of DE genes, without corresponding expression values, because their scoring methods are based only on the relative positions of the genes in the graph. Methods which use cut-offs are sensitive to the chosen threshold value, because a small change in the cut-off may drastically change the number of selected genes (Nam and Kim, 2008). As a consequence, some genes with moderate differential expression may be lost, even though they might be important players in the impacted pathways (Ben-Shaul et al., 2005). Furthermore, the genes included in the set of DE genes can vary dramatically if the selection methods are changed. Hence, the results of pathway analyses based on DE genes may be vastly different depending on both the selection method as well as the threshold value (Pan et al., 2005). On the other hand, methods which do not use a threshold are more sensitive to the noise coming from the (very many) genes that do not change much between the two phenotypes, genes that are normally eliminated by the DE selection process. An approach used to address this issue while still using all gene measurements uses the individual *p*-values of each gene (Voichiţa et al., 2012).

Among the surveyed methods, ScorePAGE, PathOlogist, NetGSA, TopologyGSA, PWEA, TAPPA, ACST, BPA, BAPA-IGGFD, and DEGraph use all genes together with their expression values as input. However, for BPA and BAPA-IGGFD^2^, the fold changes are only used to label each gene and not considered in the analysis itself. In BPA, this label is whether the gene is DE or not and in BAPA-IGGFD, the label states whether the gene is up-regulated or down-regulated. Therefore, these two methods can be categorized as using a cut-off on the input gene list. Methods that use the DE gene list and their associated values include Pathway-Guide, Pathway-Express, SPIA, and TBScore. However, the impact analysis which is the approach used by Pathway-Guide, Pathway-Express and SPIA has been recently extended to work with the set of all genes as well (Voichiţa et al., 2012), so these can now be used either with or without DE genes. Moreover, this functionality is now available as part of the Bioconductor package ROntoTools.^3^ MetaCore, TopoGSA, and EnrichNet use only the DE gene list without associated expression values. CePa is a method that has two options. It can work with either a list of DE genes, or the whole list of genes with their expression values and phenotype labels. GANPA and THINK-Back Density Analysis (DS) modify existing gene set analysis methods, such as GSEA, by calculating topology-based weights for each gene before applying the main gene set analysis method. In these methods, the gene set analysis used in the second stage uses as input the list of all genes with their expression values. However, the weighting process used in the first stage requires DE genes with their values, for GANPA, and the list of DE genes, for THINK-Back-DS.

### 2.2. Pathway data

Biological processes can be represented by different types of models. Usually pathways, such as signaling or metabolic pathways, are sets of genes and/or gene products that interact with each other in a coordinated way to accomplish a given biological function or process. A typical signaling pathway (in KEGG for instance) uses nodes to represent genes or gene products and edges to represent signals, such as activation or repression, that go from one gene to another. A typical metabolic pathway uses nodes to represent biochemical compounds and edges to represent reactions that transform one or more compound(s) into one or more other compounds. These reactions are usually carried out or controlled by enzymes, which are in turn coded by genes. Hence, in a metabolic pathway, genes or gene products are associated with edges rather than nodes, as in a signaling pathway. The immediate consequence of this difference is that many techniques cannot be applied directly on all available pathways. There are other types of biological networks that incorporate genome wide interactions between genes or proteins such as protein-protein interaction (PPI) networks. These networks are not restricted to specific biological functions. The main caveat related to PPI data is that most such data are obtained from a bait-prey laboratory assay, rather than from *in vivo* or *in vitro* studies. The fact that two proteins stick to each other in an assay performed in an artificial environment can be misleading since the two proteins may never be present at the same time in the same tissue or the same part of the cell.

The pathway data that is the input of the pathway analysis methods, generally come from a single source such as a single pathway database. In some analysis methods a second source of interaction data is used, such as a gene/protein interaction knowledge base or a genome scale network. Most of the methods use one data source. However, among the surveyed methods, MetaCore, GANPA, BAPA-IGGFD, and EnrichNet use two sources of interaction data. MetaCore uses two types of proprietary knowledge: an interaction database, as well as canonical pathways. The interaction information is protein-protein interaction data gathered from literature which is used to generate a directed global network. There is no public information regarding the details of how the MetaCore interaction network and canonical pathways are created.

Another analysis method that uses two sources of data is BAPA-IGGFD. The first source is a predefined pathway knowledge base. BAPA-IGGFD is advertised as able to analyze any pathway format; however the example in Zhao et al. (2012) is restricted to pathways from the KEGG database. The second source is an interaction knowledge base, called PrimeDB, which was created by the authors of Zhao et al. (2012), by extracting directed gene-gene interaction information from scientific publications and past experiments. PrimeDB lists potential interactions between each pair of genes and counts reported instances of activation and inhibition separately.

EnrichNet and GANPA are other methods with two input sources. They use genome-scale interaction networks in addition to predefined pathway datasets as input. For the genome-scale interaction networks, EnrichNet uses PPI networks such as STRING (Snel et al., 2000; Von Mering et al., 2003) and GANPA builds a network, called gNET, based on different types of gene/protein association databases such as PPIs, co-annotation in GO Biological Process (BP), and co-expression in large-scale gene expression microarray data.

Pathway analysis methods can use public or proprietary input sources. MetaCore, BAPA-IGGFD, and GANPA use proprietary interaction networks. All other surveyed methods use public sources. Among them, TopoGSA infers PPI networks on the fly, for human and some model organisms, from databases such as MIPS (Mewes et al., 1999), DIP (Xenarios et al., 2000), BIND (Bader et al., 2001), HPRD (Peri et al., 2004), IntAct (Hermjakob et al., 2004), and BioGRID (Stark et al., 2006). TopoGSA also accepts any kind of predefined pathways as input which it scores and compares with the constructed network.

Publicly available curated pathway databases used by the surveyed methods are KEGG (Ogata et al., 1999), NCI-PID (Schaefer et al., 2009), BioCarta (BioCarta, 2000), WikiPathways (Pico et al., 2008), PANTHER (Mi et al., 2005), and Reactome (Joshi-Tope et al., 2005). These curated knowledge bases are more reliable than protein interaction networks but do not include all known genes and their interactions. As an example, KEGG included only about 5000 human genes in signaling pathways, at the time of writing this article.

Various research groups have tried different strategies to address the challenge of modeling complex biomolecular phenomena. These efforts have lead to variation among knowledge bases, complicating the task of developing pathway analysis methods. There is currently no accepted standard for constructing pathways, and as pathway paradigms evolve to better represent the biology, pathway analysis methods evolve in parallel. Depending on the database, there may be differences in: information sources, experiment interpretation, models of molecular interactions, or boundaries of the pathways. Therefore, it is possible that pathways with the same designation and aiming to describe the same phenomena may have different topologies in different databases. As an example, one could compare the insulin signaling pathways of KEGG and BioCarta. BioCarta includes fewer nodes and emphasizes the effect of insulin on transcription, while KEGG includes transcription regulation as well as apoptosis and other biological processes. However, BioCarta includes the C-JUN transcription factor, which is missing from the KEGG representation.

**Figure 5:**
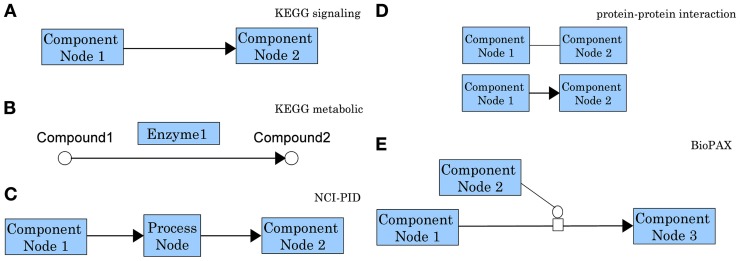
**Comparison of representative graph models for molecular interactions as used by different pathway databases.** In a KEGG signaling pathway **(A)** nodes represent genes/gene products and edges represent regulatory signals such as activation, inhibition, phosphorylation, etc. (see http://www.genome.jp/kegg/document/help_pathway.html for details). In the chemical network representation of a KEGG metabolic pathway **(B)** the nodes represent biochemical compounds and edges represent chemical reactions. These chemical reactions are performed by enzymes which are proteins encoded by genes. Hence, in contrast with the signaling pathways in which genes are associated with nodes, in a metabolic pathways genes are associated with edges. This is the main reason most methods developed for signaling pathways cannot be applied directly to metabolic pathways. In an NCI-PID signaling pathway **(C)** nodes fall in two categories: component nodes representing biomolecular components, or process nodes representing biochemical reactions or biological processes. Edges connect two biomolecular components through a biochemical reaction or a biological process. Process nodes can have 3 states: positive regulation, negative regulation, or “involved in.” (see http://pid.nci.nih.gov/userguide/network_maps.shtml for details). In a protein-protein interaction network **(D)** nodes represent proteins and the interactions among them represent physical binding. These interactions can be inferred from two-hybrid assays and they may be either undirected (top), or directed from the bait protein to the prey protein (bottom). In the Biological Pathway Exchange (BioPAX) **(E)** nodes are physical entities and edges are conversions. BioPAX entities can represent complexes, DNA, proteins, RNA, small molecules, DNA regions or RNA regions. Conversions can represent biochemical reactions complex assembly or degradation, transport or transport with biochemical reaction. This model is very generic and increasingly flexible. It provides a standard for pathway information to be available in machine readable format, therefore easy to use for pathway analysis and to exchange between pathway databases (see http://www.biopax.org/release/biopax-level3-documentation.pdf for details).

Differences in graph models for molecular interactions are particularly apparent when comparing the signaling pathways in KEGG and NCI-PID. While KEGG represents the interaction information using the directed edges themselves, NCI-PID introduces “process nodes” to model interactions (see Figure 5). Most pathway analysis methods are designed to use only one pathway graph model, which limits the user's possibilities. Developers are faced with the challenge of modifying methods to accept novel pathway databases or modifying the actual pathway graphs to conform to the method.

Pathway databases not only differ in the way that interactions are modeled, but their data are provided in different formats as well (Chuang et al., 2010). Common formats are Pathway Interaction Database eXtensible Markup Language (PID XML), KEGG Markup Language (KGML), Biological Pathway Exchange (BioPAX) Level 2 and Level 3, System Biology Markup Language (SBML), and the Biological Connection Markup Language (BCML) (Beltrame et al., 2011). The NCI provides a unified assembly of BioCarta and Reactome, as well as their in-house “NCI-Nature curated pathways,” in NCI-PID format (Schaefer et al., 2009). In order to unify pathway databases, pathway information should be provided in a common format. XML is a flexible text format with increasing use for data exchange across different systems. However, XML is very low-level and lacks standard constructs to accurately describe biological phenomena. PID XML is both human- and machine-readable, and allows a platform-independent means of exchanging PID data. The BioPAX project is an effort to unify the format and exchange of pathway data, and has incorporated independent sources such as NCI, BioCarta, Reactome, and WikiPathways, UCSC, NIH, and others (BioPAX, 2002).

The implementation of analysis methods constrains the software to accept a specific input pathway data format, while the underlying graph models in the methods are independent of the input format. Regardless of the pathway format, this must be parsed into a computer readable graph data structure before being processed. The implementation may incorporate a parser, or this may be up to the user. For instance, SPIA accepts any signaling pathway or network if it can be transformed into an adjacency matrix representing a directed graph where all nodes are components and all edges are interactions. NetGSA is similarly flexible with regard to signaling and metabolic pathways. SPIA provides KEGG signaling pathways as a set of pre-parsed adjacency matrices. The methods described in this paper may be restricted to only one pathway database, or may accept several. The corresponding databases for the surveyed methods are shown in Table 1.

**Table 1:** **Comparison of topology-based pathway analysis methods based on different criteria related to the input**.

**Method name**	**Experiment input**	**Interaction network database name**	**Year**	**References**
ScorePAGE	All genes expression	KEGG metabolic	2004	Rahnenführer et al., 2004
MetaCore^*^	DE genes list	Literature-based genome-scale interaction network; proprietary canonical pathway, genome-scale network	2004	N/A
Pathway-Express	DE genes with values, All genes expression^**^	KEGG signaling	2005	Khatri et al., 2005, Drăghici et al., 2007, Khatri et al., 2007, Voichiţa et al., 2012
TAPPA	All genes expression	KEGG metabolic	2007	Gao and Wang, 2007
PathOlogist	All genes expression	KEGG	2007	Efroni et al., 2007
Pathway-Guide^*^	DE genes with fold change (FC) values, DE genes list, All genes with values, DE genes with FCs and *p*-values	KEGG signaling, REACTOME, NCI, BioCarta	2009	N/A
SPIA	DE genes with values	KEGG signaling	2009	Tarca et al., 2009
NetGSA	All genes expression	KEGG signaling	2009	Shojaie and Michailidis, 2009,Shojaie and Michailidis, 2010
PWEA	All genes expression	YeastNet	2010	Hung et al., 2010
TopoGSA	DE genes list	Genome-scale PPI network, KEGG	2010	Glaab et al., 2010
PARADIGM	All genes expression, copy number, proteins level	Constructed PPI networks from MIPS, DIP, BIND, HPRD, IntAct, and BioGRID	2010	Vaske et al., 2010
TopologyGSA	All genes expression	NCI-PID	2010	Massa et al., 2010
DEGraph	All genes expression	KEGG	2010	Jacob et al., 2010
MetPA	DE metabolites with values	KEGG metabolic	2010	Xia and Wishart, 2010
BPA	All genes expression - with cut-off	NCI-PID	2011	Isci et al., 2011
GANPA	DE genes with values, All genes expression	Genome-scale PPI network, KEGG, REACTOME, NCI-PID, HumanCyc	2011	Fang et al., 2011
BAPA-IGGFD	All genes expression - with cut-off	Literature-based gene-gene interaction database, KEGG, WikiPathways, REACTOME, MSigDB, GO BP, PANTHER; constructed gene association network from PPIs; co-annotation in GO Biological Process (BP); and co-expression in microarray data	2012	Zhao et al., 2012
CePa	DE genes list / All genes expression	NCI-PID	2012	Gu et al., 2012
THINK-Back-DS	DE genes with values, All genes expression	KEGG, PANTHER, BioCarta, REACTOME, GenMAPP	2012	Farfán et al., 2012
TBScore	DE genes with values	KEGG signaling	2012	Ibrahim et al., 2012
ACST	All genes expression	KEGG signaling	2012	Mieczkowski et al., 2012
EnrichNet	DE genes list	Genome-scale PPI network, KEGG, BioCarta, WikiPathways, REACTOME, NCI-PID, InterPro, GO with STRING 9.0	2012	Glaab et al., 2012

* *commercial methods*;

** released in 2013 as part of ROntoTools.

(http://www.bioconductor.org/packages/release/bioc/html/ROntoTools.html)

N/A, No publication available. Experiment input describes the type of experiment data input required by the method. The meaning of each term is as follows: “DE genes with values/DE metabolites with values” represents the list of differentially expressed (DE) genes or metabolites with their fold-change value or t-statistics. Sometimes this list is accompanied by the list of total genes monitored in the experiment; “DE genes list” represents a list of selected genes, usually DE genes (this is just a list of IDs, without associated fold-changes). “All genes expression” represents the list of all genes in all samples together with their expression values. Some methods require all genes, but then perform the analysis using a flag for the DE genes - these are marked as “with cut-off.” Some methods use one type of input in a gene weighting stage while using another type of input to assess the pathway significance. Interaction network type and database name is the input knowledge source for the analysis method and the databases proposed by the software. Some of the methods can use any pathway, but provide parsed data for the pathway databases listed here. “Pathway” refers to any kind of signaling or metabolic pathway or gene regulatory network. “Genome scale interaction network” refers to interaction networks constructed from protein interactions or co-annotation from GO databases, literature, or co-expression inferred from existing microarray experiments. “Constructed network” means that the analysis method uses pathways created by its authors rather than pathways from a reference database. Year denotes the year of the first published paper describing the method. References denotes the first published paper describing the method.

## 3. Mathematical models

For topology-based pathway analysis methods, the mathematical model describes how the graph and the experiment data are processed to compute a score for each pathway. The score quantifies the significance of changes in a (sub)pathway between the two phenotypes. This score may be a statistical significance or other non-statistical method-specific metric. The diversity of current topological based pathway analysis methods reflects the variety of mathematical models available for graphs. The output is typically a list of ranked (sub)pathways.

### 3.1. Graph models

Two major graph models are used to represent biological networks and pathways. The first model, hereon referred to as “single-type,” allows only one type of node, the biological component (i.e., a gene or protein), with edges representing molecular interactions occurring between the nodes (e.g., Figure 5A). In contrast, the second graph model, hereon referred to as “multi-type,” allows multiple type of nodes, such as components and interactions (e.g., Figure 5C). Multi-type graph models are more complex than single-type, but they capture more pathway characteristics. For example, single-type models are limited when trying to describe “all” and “any” relations between multiple components that are involved in the same interaction. Bipartite graphs, which contain two types of nodes and allow connection only between nodes of different types, are a particular case of multi-type graph models.

In most databases, pathways use the single-type graph model and the signaling and metabolic pathways from databases such KEGG and BioCarta are good examples. In signaling pathways, nodes are genes and edges describe various molecular interactions, which include activation/transcription/positive regulation, repression/blockage/negative regulation, (de)phosphorylation, binding/association. Metabolic pathways can be represented as either chemical networks or protein networks. In the chemical network representation, nodes are metabolites and edges are enzymes and/or substrates that catalyze the chemical reactions. In the protein network, the representation is reversed; nodes are enzymes and edges are metabolites. Among the surveyed methods which work with metabolic pathways only MetPA uses biochemical networks from KEGG. ScorePAGE and TAPPA use protein networks. Nevertheless, the most popular representation of metabolic pathways in public databases is the chemical network. In KEGG and BioCarta, the majority of edges in both metabolic and signaling pathways are directed, but binding between compounds is represented by undirected edges.

Protein-protein interaction (PPI) networks, constructed from interaction databases, use a single-type graph model. The nodes represent proteins and the edges depict their association/binding. Sometimes the edges are undirected, while some other times, the edges are directed to describe which protein was used as the bait and which one acted as the prey.

Reactome and NCI-PID are databases that use a bipartite graph model to represent pathways. Genes, metabolites, or molecular complexes are represented as component nodes, while interaction nodes define the chemical reactions or molecular processes that occur between the input and output component nodes. The edges, which connect a component node to an interaction node, specify the component's type of contribution to the reaction. These can be positive or negative regulation, among others.

The majority of analysis methods surveyed here use a single-type graph model. Some apply the analysis on a directed or un-directed single-type network built using the input pathway, while others transform the pathways into graphs with specific characteristics. An example of the later is TopologyGSA, which transforms the directed input pathway into an undirected decomposable graph, that has the advantage of being easily broken down into separate modules (Lauritzen, 1996). In this method, decomposable graphs are used to find “important” submodules - those which drive the changes across the whole pathway. For each pathway, TopologyGSA creates an undirected moral graph^4^ from the underlying directed acyclic graph (DAG) by connecting the parents of each child and removing the edge direction. The moral graph is then used to test the hypothesis that the underlying network is changed significantly between the two phenotypes. If the the research hypothesis is rejected, a decomposable/triangulated graph is generated from the moral graph by adding new edges. This graph is broken into the maximal possible submodules and the hypothesis is re-tested on each of them.

BPA is another method that implements pathway graph pre-processing. This method uses Bayesian networks to represent biological pathways. In Bayesian networks, random variables are assigned to each node of a DAG network and the edges represent the conditional dependencies between nodes. Before assigning the random variables, the pathway graph is checked for cycles. If the graph is not a DAG, Spirtes' method (Spirtes, 1995) is used to remove the cycles while the (in)dependency rules in the initial pathway graph are preserved.

Another example is BAPA-IGGFD, which is a method that simplifies pathway graphs by removing any edge representing interactions other than activation and inhibition. In addition, the pathways are pruned keeping only elements from three categories: signal receptors (including ligands) are at the beginning, transcription factors are usually at the end, and their direct regulators are in the middle. This pre-processing is motivated by noise reduction in the final scoring of genes that have a less important functional role in the pathway or belong to multiple pathways where they play different roles. (Zhao et al., 2012) includes only an intuitive high-level description of this process is presented, without a detailed algorithm.

CePa uses a different method to modify the input pathways before the analysis. The NCI knowledge base is used as a source of NCI-Nature, BioCarta, Reactome, and KEGG pathways, which are provided in PID or short NCI-PID format. The pathway data is organized in the form of multi-type graphs, which are used to generate directed single-type graphs, where each node can represent one or multiple genes. A node in the generated graph is considered to be DE if any of its gene components is DE. Unfortunately, the details of how the original pathways are parsed to generate the new networks are not provided by the authors of CePa.

PathOlogist and PARADIGM are the two surveyed methods that use multi-type graph models. PathOlogist uses a bipartite graph model with component and interaction nodes. PARADIGM, conceptually motivated by the central dogma of molecular biology, takes a pathway graph as input and converts it into a more detailed graph, where each component node is replaced by several more specific nodes: biological entity nodes, interaction nodes, and nodes containing observed experiment data. The observed experiment nodes could in principle contain gene expression and copy number information. Biological entity nodes are DNA, mRNA, protein, and active protein. The interaction nodes are transcription, translation, or protein activation, among others. Biological entity and interaction node values are derived from these data and specify the probability of the node being active. These are the hidden states of the model.

### 3.2. Scoring methods

**Figure 6:**
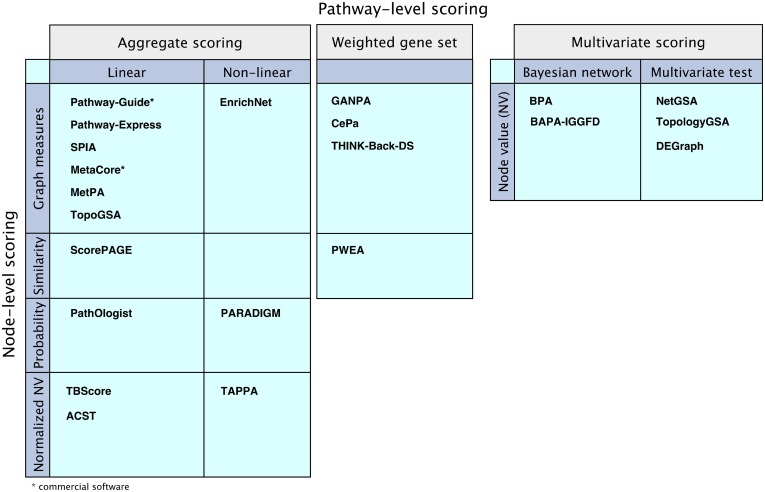
**Comparison of the mathematical models of the surveyed pathway analysis methods.** “Aggregate scoring” and “Weighted gene set” panels show methods that perform node-level scoring followed by pathway-level scoring performed either as an aggregation of the node scores or as a weighted gene set analysis, using the node scores as weights. The methods are divided according to their node-level scoring methods: graph measure techniques, similarity measurement techniques, probabilistic models, or using normalized node values based on node value and/or pathway structure. The “Multivariate scoring” methods use multivariate scoring models without node-level scoring. They use node values to directly compute a pathway score using Bayesian networks or applying multivariate hypothesis tests.

The goal of the scoring method is to compute a score for each pathway based on the graph model, resulting in a ranked list of pathways or sub-pathways. There are a variety of approaches to quantify the changes in a pathway. Some of the analysis methods use a hierarchically aggregated scoring algorithm, where on the first level, a score is calculated and assigned to each node or pair of nodes (component and/or interaction). On the second level, these scores are aggregated to compute the score of the pathway. On the last level, the statistical significance of the pathway score is assessed using univariate hypothesis testing. Another approach, used by BPA, BAPA-IGGFD, NetGSA, TopologyGSA, and DEGraph, assigns a random variable to each node and a multivariate probability distribution is calculated for each pathway. The output score can be calculated in two ways. One way is to use multivariate hypothesis testing to assess the statistical significance of changes in the pathway distribution between the two phenotypes. The other way is to estimate the distribution parameters based on the Bayesian network model and use this distribution to compute a probabilistic score to measure the changes. In this section, we provide details regarding the scoring algorithms of the surveyed methods. See Figure 6 for scoring algorithms categories.

#### 3.2.1. Hierarchically aggregated scoring algorithms

**Figure 7:**
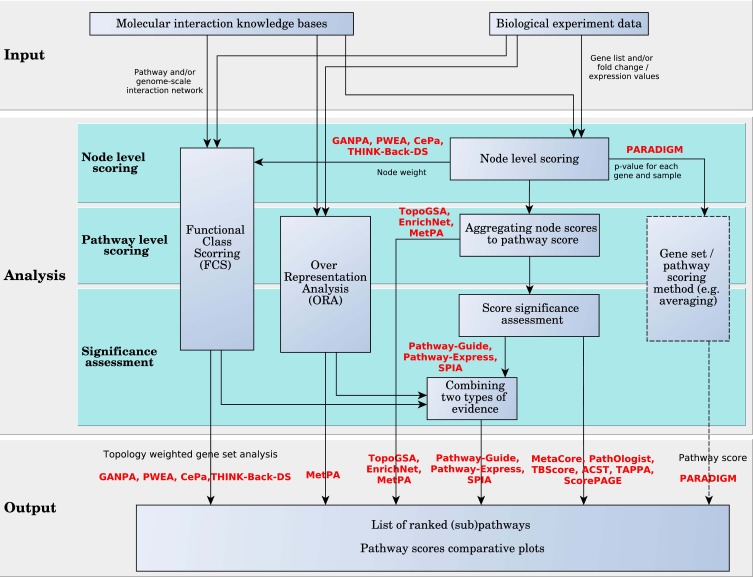
**Diagram of pathway analysis scoring approach for hierarchically aggregated scoring algorithms.** The box with the dashed border indicates that the user can choose these options, but are not offered by the method implementation.

These analysis approaches are detailed in Figure 7. In this figure, the analysis is divided into three levels: node-level scoring, pathway-level scoring and significance assessment. All methods compute node level scores. One or both remaining levels may be skipped by certain approaches. PARADIGM is the only one that provides as direct output the node scores, rather than the pathway scores. These scores can be input into a gene set or pathway analysis algorithm, or a simple averaging function can be used to score the pathways and rank them, as in Vaske et al. (2010). The rest of the methods go on to the second level where the scores of the pathways are calculated. Some methods stop at the second level, outputting the whole list of ranked pathways without evaluating their statistical significance, which is done by the remaining methods on the next level.

***Node Level Scoring.*** Here we categorize and describe the surveyed methods based on their node level scoring model. Most of the surveyed analysis methods incorporate pathway topology information in the node scores. There are methods such as TAPPA and ACST that incorporate this information in the pathway scores. In TAPPA, the score of each node is the square root of the normalized log gene expressions (node value). ACST calculates the node level score using a sign statistic. The sign reflects the direction of the gene expression change between the phenotypes under study. This statistic can be a represented by a *t*-value or the log fold change of the gene expression. The statistic is standardized using a local mean and standard deviation.

The rest of the analysis algorithms use a variety of approaches to incorporate topology in the node level scores. We categorize them into methods that use graph measures (centrality), similarity measures, and probabilistic graphical models. TBScore is an exception that can not fall into either of these groups. TBScore weights the pathway DE genes based on their log fold change and the number of distinct DE genes directly downstream of them, using a depth-first search algorithm.

MetaCore, Pathway-Guide, Pathway-Express, SPIA, TopoGSA, CePa, EnrichNet, MetPA, THINK-BACK-DS, and GANPA use centrality measures or a variation of these measures to score nodes in a given pathway. Centrality measures describe the importance of a node relative to all other nodes in a network. There are several centrality measures that can be applied to networks of genes and their interactions and these are degree centrality, closeness, betweenness, and eigenvector centrality. Degree centrality accounts for the number of directed edges that enter and leave each node. Closeness sums the shortest distance from each node to all other nodes in the network. Node betweenness adds a layer of complexity to closeness; it measures the importance of a node according to the number of shortest paths that pass through it. Eigenvector centrality uses the network adjacency matrix of a graph to determine a dominant eigenvector; each element of this vector is a score for the corresponding node. Thus, each score is influenced by the scores of neighboring nodes. In the case of directed graphs, a node that has many downstream genes has more influence and receives a higher score.

In MetaCore, a measure similar to node betweenness is used to score genes. There is no peer-reviewed paper publicly available describing the details of the MetaCore pathway analysis method. We used the study by Dezső et al. (2009) to uncover some of these details. In the method by Dezső et al., the DE gene list is overlapped with a global genome scale network containing all the interactions in the MetaCore knowledge base. A network, which is called condition specific shortest-path network (CSSPN), is built based on this overlap. In addition to DE genes, all genes which are on shortest paths that connect them in the global network are included in the CSSPN. For each pair of genes (*g*_*i*_, *g*_*j*_), where *g*_*i*_ is in the CSSPN and *g*_*j*_ is in the set of DE genes, two parameters *N*_*ij*_ and *K*_*ij*_ are computed. *N*_*ij*_ is the number of times *g*_*i*_ is part of the shortest-paths in the global network between *g*_*j*_ and every other gene in the CSSPN. *K*_*ij*_ is the number of times *g*_*i*_ is part of the shortest-paths in the global network between *g*_*j*_ and every other gene in the set of DE genes. It is assumed that the probability to observe these numbers just by chance, given the two sets of genes, the global network which is of size *N* and the DE genes which is of size *K*, follows a hypergeometric distribution. Based on this distribution, *K p*-values are computed for each gene in the CSSPN and the minimum of these *p*-values is selected as the gene score. Using a predefined threshold on the false discovery rate (FDR) correction of the node scores, a subset of the CSSPN genes is selected. Further processing, in the pathway level scoring, is applied to this list of selected genes.

Pathway-Guide, Pathway-Express, and SPIA use a perturbation factor, which takes into consideration the magnitude of all gene expression changes, the type of each gene, the direction and type of all gene interactions, as well as the efficiency with which the perturbation of each gene propagates to the downstream genes. The impact analysis models the flow of the signals in the pathways. In essence, the impact factor falls into the eigenvector centrality category of node scoring approaches. Although all three methods use the same impact analysis approach, there are slight differences between them. Pathway-Guide scores the pathways based on the impact factor as briefly described above In SPIA, the amount of differential expression is subtracted from the perturbation score of each node to focus on the amount of perturbation accumulated at any given node in order to separate the influence of experiment data and topology. Pathway-Guide is also able to exploit the *p*-values associated with each gene, as well as identify coherent perturbation cascades that represent putative mechanisms that explain all measured changes. All three methods combine the perturbation evidence with a classical enrichment (e.g., hypergeometric), or functional class scoring (e.g., GSEA) to calculate a global *p*-value. This corresponds to the joint probability of a pathway having the measured amount of perturbation, as well as the observed number of DE genes just by chance. TBscore has an interestingly similar approach in capturing the pathway perturbation, with the difference that DE genes with more connected downstream DE genes are considered more significant.

TopoGSA extracts a network from databases of protein interactions given a list of genes/proteins of interest. All four types of centrality measures and a fifth measure, called a “clustering coefficient” (Watts and Strogatz, 1998) are used to score the nodes in this network. Then, each predefined pathway from a selected dataset is also scored using the same five measures, independently of the extracted network. Comparing the summarized node scores for each pathway with node scores from the extracted network allows the pathways to be ranked.

In CePa, node weights are computed using five centrality-based measures, and there is an extra case where all the node weights are assumed to be equal. The five measures are: in-degree, out-degree, betweenness, in-reach (length of longest shortest path that starts from the node), and out-reach (length of longest shortest path that ends at the node). CePa offers two options to assess the significance of pathways. One is based on the hypergeometric analysis using only node weights. The second is based on enrichment analysis and in addition to node weights, node scores are needed. Node scores are computed using a t-statistic. Pathway graphs in CePa can contain nodes representing one or multiple genes. In the case of single-gene nodes, the score is calculated based on the expression value of the corresponding gene. In the case of multi-gene nodes, the node score is the largest principal component of the expression values of the genes in the node.

EnrichNet uses a score similar to centrality closeness measures. This method calculates two distance vectors. The first vector contains distances between a list of input genes and a predefined pathway/gene set. The second vector contains the distance between the same input gene list and a background global set containing all pathways. A node score is computed as the distance between the node and all DE genes using a random walk with restart algorithm (Yin et al., 2010) through a genome scale molecular interaction network. The interaction network is represented by its weighted adjacency matrix, where weights are interaction strengths provided by the input knowledge base.

MetPA allows the user to select either the node betweenness or the out-node degree centrality measure for the node score. GANPA (Fang et al., 2011) uses the node degree measure as a weight or score for the gene. THINK-Back-DS uses a measure similar to closeness called density score to emphasize the DE genes which are in tight clusters.

ScorePAGE and PWEA use similarity measures in their node level scoring. Similarity measures estimate the coexpression, behavioral similarity, or co-regulation of pairs of components. Their values can be correlation coefficients, covariances, or dot products of the gene expression profile across time or sample. In these methods, the pathways with clusters of highly correlated genes are considered more significant. At the node level, a score is assigned to each pair of nodes in the network which is the ratio of one similarity measure over the shortest path distance between these nodes. Thus, the topology information is captured in the node score by incorporating the shortest path distance of the pair. In ScorePAGE, the correlation coefficient, covariance, or dot product is calculated for all gene pairs across their samples. PWEA uses the correlation coefficient to score node pairs. In this method, a score, called “Topological Influence Factor,” or TIF, is assigned to each gene by exponentially averaging the score of all pairs that include the gene. As a consequence, a node involved in tight clusters of highly correlated genes has a higher score.

PARADIGM and PathOlogist incorporate the topology in the node level scoring using a probabilistic graphical model. In this model, nodes are random variables, and edges define the conditional dependency of the nodes they link. PARADIGM takes observed experiment data and calculates scores for all component nodes, in both observed and hidden states, from the detailed network created by the method based on the input pathway. For each node score, a positive or negative value denotes how likely it is for the node to be active or inactive, respectively. The scores are calculated to maximize the occurrence probability of the observed values. A *p*-value is associated with each score of each sample such that each node can be tagged as significantly active, significantly inactive, or not-significant. For each network, a matrix of *p*-values is output, in which columns are samples, and rows are component nodes.

PathOlogist is also based on a probabilistic graphical model. This method estimates the parameters of one or two distributions related to the up and/or down regulation of each gene using its expression values across all samples. These distributions are used to assign a probability score to each gene in each sample, denoting how likely it is for the gene to be highly expressed. The method assigns two different scores to interaction nodes: (i) the “activity score,” which is the probability that the parents of an interaction node (which are component nodes) are highly expressed, and (ii) the “consistency score,” which is the probability that the interaction node is active and its children are expressed or inactive with unexpressed children.

***Pathway Scoring Level.*** In the following, we describe how node scores are used to compute pathway scores. Many of the surveyed methods aggregate node level statistics to pathway level statistics using linear functions such as averaging or summation. The methods that use linear aggregation in this level of the analysis are: TopoGSA, MetaCore, MetPA, ScorePAGE, TBScore, ACST, PathOlogist, Pathway-Guide, Pathway-Express, and SPIA. The rest of the methods either use a nonlinear function to aggregate the node scores to pathway scores, like TAPPA, PARADIGM, and EnrichNet, or apply a gene set analysis method on the node scores, like GANPA, CePa, THINK-Back-DS, and PWEA.

In MetaCore, important genes are selected in the gene level scoring based on the list of DE genes and the network topology. At the pathway level, this method assumes that the number of selected genes that fall on a pathway is the pathway score and follows the hypergeometric distribution.

In TAPPA, the pathway score for each sample is a weighted sum of the product of all node pair scores in the pathway. The weight coefficient is 0 when there is no edge between a pair. For any connected node pair the weight is a sign function, which represents joint up- or down-regulation of the pair.

In ACST, pathway scores are calculated based on the position of node (gene) clusters for which the interaction types match the up- or down-regulation of genes. This uses the same concept of coherent signals used by Pathway-Guide. An edge (interaction) between 2 components in a pathway is called consistent if either (i) the pair has an inhibition interaction, and the directions of differential expression of the components is opposite, or (ii) the pair has an activation interaction, and the direction of differential expression of the components is the same. All other interaction types are ignored. Maximal consistent graphs are defined as maximal sub-networks of the pathway in which all interactions are consistent. The score of each maximal consistent sub-graph is the summation of all node scores. The pathway score is the sum of the scores of all its maximal consistent sub-graphs. Node scores are t-statistics normalized by the distance from the sub-graph to the leaves of the pathway graph. The authors argue that the consistent sub-graphs close to the leaves of the pathway have a greater impact on the score of pathway rather than the clusters from the beginning of the pathway. This is somewhat different from the approach that Pathway-Guide, Pathway-Express, and SPIA follow. Although in these methods there is no explicit weighting based on the up- or down-stream position of a gene in a pathway, just because the perturbation of one gene is propagated following the signals described by the pathway, the perturbation of a gene somewhat near the entry point in a pathway will have more impact than the same amount of perturbation for a gene somewhere downstream on the pathway. Only time and additional testing will tell which of the two approaches manages to capture better the biological phenomena.

In EnrichNet, pathway scores measure the difference of the node score distribution for a pathway and a background network/gene set which consists of all pathways. At the node level, the distance of all DE genes to the pathway is measured and summarized as a distance distribution. The method assumes that the most relevant pathway is the one with the greatest difference between the pathway node score distribution and the background score distribution. The difference between the two distributions is measured by the weighted averaging of the difference between the two discretized and normalized distributions. The averaging method down-weights the higher distance nodes and emphasizes the lower distance ones.

Methods such as Pathway-Guide, Pathway-Express, SPIA, and MetPA use two types of analysis to score the pathways. For each pathway, these methods calculate both a topology based score and a *p*-value from a gene set enrichment analysis measure, such as Fisher's exact test, hypergeometric, or GlobalAncova. Pathway-Guide, Pathway-Express, and SPIA use the joint probability of observing the pathway perturbation, as well as the gene enrichment on a given pathway (Drăghici et al., 2007). This model effectively combines the topology-based pathway score with the one based on enrichment to provide a single global pathway score. MetPA (Xia and Wishart, 2010) also looks at both enrichment and topology, but does not assess the significance of the topology-based pathway scores and does not combine the two scores, and thus lacks a unique significance ranking. The most impacted pathways in MetPA are those with higher scores in both measures. It is not clear how to treat a trade-off between the two types of significance.

The pathway scoring techniques described so far in this section incorporate in-house analysis methods. A different direction is to design scoring techniques that incorporate existing gene set analysis methods, such as GSEA (Subramanian et al., 2005), GSA (Efron and Tibshirani, 2007), or LRPath (Sartor et al., 2009). Pathway-level scores can be calculated using node scores which represent the topology characteristic of the pathway as weight adjustments to a gene set analysis method. PWEA, GANPA, THINK-Back-DS, and CePa use this approach and we refer to them as weighted gene set analysis methods. GSEA calculates the correlation coefficient of phenotype with gene expression (*CC*), GSA and LRPath use the *t*-test statistic in the computation of the node score. To compute the pathway score, PWEA adjusts the *CC* exponent of 0 or 1 in GSEA to *CC*^*TIF*+1^, where *TIF* is the node weight described above. The node weights calculated by GANPA, THINK-Back-DS, and CePa are used to adjust *CC* or the t-statistic by multiplication, *node weight* × *CC* or *node weight* × *t* − *statistic*. In CePa there is another option to use a hypergeometric analysis to calculate pathway scores. In this method, the node weights of DE nodes are summed up to the pathway level.

Some methods such as Pathway-Guide, Pathway-Express, SPIA, and ROntoTools offer the flexibility to integrate in the analysis any type of enrichment technique. Thus, the *p*-values provided by techniques such as GSEA, GSA, or PADOG (Tarca et al., 2012) can be used instead of the *p*-values provided by simpler models such as hypergeometric.

***Pathway Significance Assessment.*** Pathway scores are intended to provide information regarding the amount of change incurred by the pathway between two phenotypes. However, the amount of change is not meaningful by itself since any amount of change can take place just by chance (i.e., the amount of change is only the effect size). An assessment of the *significance* of the measured changes is thus required, and should be done by analysis methods in the pathway significance assessment level.

Methods such as TopoGSA, MetPA, and EnrichNet, will output scores without any significance assessment, leaving it up to the user to interpret the results. This is problematic because the user does not have any instrument to help distinguish between changes due to noise or random causes, and meaningful changes, unlikely to occur just by chance and therefore, possibly related to the phenotype. The rest of the analysis methods perform a hypothesis testing for each pathway. The null hypothesis is that the value of the observed statistic is due to random noise or chance alone. The research hypothesis is that the observed values are substantial enough that they are potentially related to the phenotype. A *p*-value for calculated score is then computed and a user-defined threshold on the *p*-value is used to decide whether the the null hypothesis can be rejected or not for each pathway. Finally, a correction for multiple comparisons should be performed.

Typically, pathway analysis methods compute one score per pathway. However, methods such as PathOlogist and TAPPA compute the pathway score considering each sample separately. Therefore, for each pathway there is a population of scores that can be analyzed. This population combined with different sample features can provide various feature-specific analyses. There are two cases to be considered based on the qualitative or quantitative nature of the sample feature values. In the first case the sample feature is qualitative with binary values. For example, when samples are tagged corresponding to the two phenotypes, the significance assessment is done by testing whether the score distributions are the same in the two groups using two-sample rank-sum tests, such as the Mann–Whitney *U*-test. If the number of samples is high enough, the score distributions can be assumed to be normal. The null hypothesis here is that the two normal distributions have equal means and variances, the research hypothesis is that they are different. In the second case the sample feature is quantitative with continuous values. Two ways to identify significant pathways are implemented in this case. One way is to partition pathway scores into a known number of clusters, for example two, using k-means clustering. Cumulative distributions are calculated for each of the two classes. A logrank test (Mantel, 1966), which is a non-parametric statistical test, can be performed to evaluate whether the behavior of the variable is same in the two groups. Significant pathways are those that can be used to divide samples into groups with different characteristics. Another way to identify significant pathways in the case of continuous sample feature values is to find pathways whose scores are linearly correlated with the values of the feature. The null hypothesis in this case is that the correlation is zero, and a *t*-test is used.

For methods that calculate one score per pathway, the distribution of this score under the null hypothesis can be constructed and compared to the observed. However, there are often too few samples to calculate this distribution, so it is assumed that the distribution is known. For example, in MetaCore and many other techniques, when the pathway score is the number of DE nodes that fall on the pathway, the distribution is assumed to be hypergeometric. However, the hypergeometric distribution assumes that the variables (genes in this case) are independent, which is incorrect, as witnessed by the fact that the pathway graph structure itself is designed to reflect the specific ways in which the genes influence each other. Another approach to identify the distribution is to use statistical techniques such as the bootstrap method (Efron, 1979). Bootstrapping can be done either at the sample level, by permuting the sample labels, or at gene set level, by permuting the the values assigned to the genes in the set.

To create the score distribution under the null hypothesis, Pathway-Guide, Pathway-Express, and SPIA methods use bootstrapping at the gene set level. For these methods, samples are drawn from the distribution of all DE genes and assigned to a gene set which is different from the DE gene set but with equal number. The pathway score is computed assuming the new gene set as a decoy DE gene set. This procedure is repeated for a number of iterations. The scores resulting from these iterations estimate the distribution, which is then used to compute a *p*-value, and a pathway score is obtained by combining the gene set enrichment evidence with the topology-based *p*-value and applying Fisher's exact test. The final score is the FDR-adjusted *p*-value.

TBScore, the hypergeometric extension of CePa, and ACST calculate *p*-values using bootstrapping at the sample level by permuting the labels of the samples of the two phenotypes. In TBScore and CePa, an iterative procedure is then used to estimate the pathway score distribution under the null hypothesis. Correction for multiple comparison, again FDR, is used to compute the final pathway *p*-values. In ACST, after *p*-values are computed, a statistical technique called “resampling-based point estimator” is used to estimate the FDRs associated with the predefined threshold.

Weighted gene set methods surveyed here, PWEA, GANPA, the enrichment analysis extension implemented by CePa, and THINK-Back-DS, focus on providing a biologically meaningful topology-based adjustment to existing gene set analysis methods. Therefore the statistical assessment of pathway significance is provided by the already developed methods among which the most popular is GSEA (see Figure 7).

#### 3.2.2. Multivariate scoring algorithms

**Figure 8:**
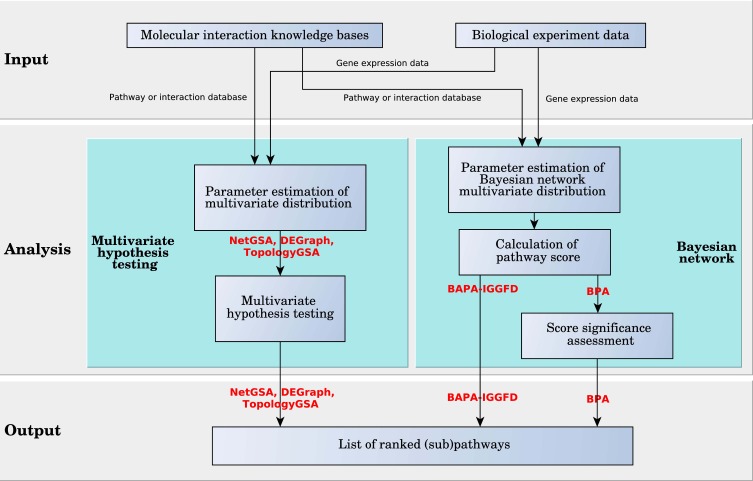
**Diagram of pathway analysis scoring approaches for multivariate scoring algorithms**.

Multivariate scoring analysis methods mostly use multivariate probability distributions to score pathways and can be grouped in two categories. Methods in the first category use multivariate hypothesis testing, while methods in the second category are based on Bayesian network (see Figure 8).

NetGSA, TopologyGSA, and DEGraph are methods based on multivariate hypothesis testing. These analysis methods assume the vectors of gene expression values in each (sub)pathway are random vectors with multivariate normal distributions. The network topology information is stored in the covariance matrix of the corresponding distribution. For a network, if the two distributions of the gene expression vectors corresponding to the two phenotypes are significantly different, the network is assumed to be significantly impacted when comparing the two phenotypes. The significance assessment is done by a multivariate hypothesis test. The definition of the null hypothesis for the statistical tests and the techniques to calculate the parameters of the distributions are the main differences between these three analysis methods.

In NetGSA, it is assumed that the expression level of the genes (nodes in the network) obtained from experiments are correlated because of the interactions between them. In other words, the edges (interactions) of a graph (pathway) imply correlations. In order to compute the distribution parameters, the method defines a set of latent variables, which are the uncorrelated gene expressions. The input correlated gene expression vector can be written in the form of the product of the vector of latent variables and the influence matrix. This matrix consists of the weights assigned to each edge measuring the strength of the interaction between two genes. The influence matrix and other parameters for the two phenotypes are computed based on linear mixed model theory (McLean et al., 1991). The proposed hypothesis test, in this method, is to check whether a linear combination of the mean of the latent variables, called contrast vector, for the two cases are equal. The proposed contrast vector is computed based on the influence matrix and it is proved that the result includes the effects of all nodes inside a chosen network and excludes any outside effects, such as the correlation.

In TopologyGSA, the directed graph is converted into a moral undirected graph, detailed in Section 3.1. The covariance matrices for each of the two phenotypes are estimated using the Iterative Proportional Scaling (IPS) algorithm (Lauritzen, 1996) on the sample covariance for all pairs of genes. The two matrices are defined such that their inverses have zero elements corresponding to the missing edges. A set of two hypothesis tests are applied to compute the statistical significance of the impact on a given graph. The first test checks whether the concentration matrices, i.e., the inverses of the covariance matrices, in the two cases are equal. If this hypothesis is rejected, the graph is broken into the maximal possible submodules, and the hypothesis is retested on each. Based on the equality of concentration matrices, different statistical techniques are used in the second hypothesis test. The second test checks the significance of the influence of the graphs based on the equality of the means of the distributions.

DEGraph finds significant (sub)pathways by using a modified multivariate Hotelling T^2^-test hypothesis. The modification incorporates the topology of the network. The difference, referred to as shift, between the mean vectors of gene expression distributions corresponding to the two phenotypes is smoothed. A shift vector is defined to be smooth if the shift values of every two connected nodes are similar. The process of smoothing is done by removing the high frequency shift values according to the topology of the network. This is achieved by filtering the shift by preserving only the first few components of the graph-Fourier basis of the shift vector. The graph-Fourier in DEGraph is applied by spectral analysis of the graph Laplacian (Chung, 1997), which resembles the Fourier decomposition of a function. The smoothed shift vector is used in the Hotelling T^2^-test to assess the statistical significance of a network. DEGraph also provides an algorithm that allows the exhaustive testing of all the sub-networks of the original network using a branch and bound algorithm.

BPA and BAPA-IGGFD are two methods based on Bayesian networks. In a Bayesian network, which is a special case of probabilistic graphical models, a random variable is assigned to each node of a directed acyclic (DAG) graph. The edges in the graph represent the conditional probabilities between nodes, so that the children are independent from each other and the rest of the graph when conditioned on the parents. In BPA, the value of the Bayesian random variable assigned to each node captures the state of a gene (DE or not). In contrast, in BAPA-IGGFD each random variable assigned to an edge is the probability that up or down regulation of the genes at both ends of an interaction are concordant with the type of interaction which can be activation or inhibition. In both BPA and BAPA-IGGFD, each random variable is assumed to follow a binomial distribution whose probability of success follows a beta distribution. However, these two methods use different approaches in representing the multivariate distribution of the corresponding random vector. BPA assumes that the random vector has a multinomial distribution, which is the generalization of the binomial distribution. In this case, the vector of the success probability follows the Dirichlet distribution, which is the multivariate extension of the beta distribution. Conversely, BAPA-IGGFD assumes the random variables are independent, therefore the multivariate distributions are calculated by multiplying the distributions of the random variables in the vector. It is worth mentioning that the assumption of independence in BAPA-IGGFD is contradicted by evidence, specifically in the case of edges that share nodes.

In BPA a discretized fold change profile is calculated for each gene. This represents the list of fold changes between every ordered pair of gene expression samples. The pair elements come from each of the groups corresponding to the two phenotypes. These fold changes are discretized such that genes with values higher than 2 or lower than 0.5 are considered differentially expressed and the others are considered to have negligible changes. This profile is used as the observed data for the Bayesian network model. In BPA, given a set of parameters (success probabilities), the likelihood of observing a specific profile on the Bayesian network is assumed to have a multinomial distribution. Using the Bayes rule, the probability of observing the given profile without any assumption on the parameters is calculated. The parameters of the distributions are learned from the input data (Neapolitan, 2004). The network topology is incorporated in the distribution parameters and computation method by assuming that knowing the values of the parents' random variables, the children random variables are independent of the rest of the graph. A hypothesis testing is performed using the null hypothesis that the probability of seeing the observed data is the result of chance. Specifically, a set of observed data is generated in the bootstrapping analysis and its probability is compared with the the original observed data. The null distribution is approximated through randomization via bootstrapping. This randomization targets the structure of the Baeysian network (i.e., the relation between its nodes), which is more relevant than a simple bootstrapping in this case. Sampling with replacement is used when generating random data. An upper-tailed test is performed, with the *p*-value estimated by the percentage of random scores higher than the observed one. The process of generating the randomized samples is done by bootstrapping. A new fold change profile is generated by sampling with replacement from the original fold change profile.

In BAPA-IGGFD, based on the value of the fold change, discretized values of 0 or 1, corresponding to up- or down-regulation, are assigned to each node of the Bayesian network. For each predefined pathway, a vector of probabilities is computed as follows: (1) ${\bar{\theta }}_{i}$ for any parent-less gene *g*_*i*_ is the probability of *g*_*i*_ being up-regulated, (2) θ_*i*|*j*_ for any gene *g*_*i*_ which has an activator parent *g*_*j*_ is the probability that both genes are coherent in being up-regulated or down-regulated, and (3) ϕ_*i*|*j*_ for any gene *g*_*i*_ which has an inhibitor parent *g*_*j*_ is the probability that the state of up or down regulation of the genes are opposite. The vector can be summarized as θ = ({${\bar{\theta }}_{i}$|∀*g*_*i*_ is parent-less}, {θ_*i*|*j*_|∀*g*_*i*_ has an *activator* parent}, {ϕ_*i*|*j*_|∀*g*_*j*_ has an inhibitor parent}) which is called the parameter vector of the pathway. Each of these parameters are assumed to be independent from each other and follow the beta distribution both prior observing the microarray data and after its observation. The multivariate joint distributions of the parameter vector prior and posterior of the data observation are compared using symmetric Kullback-Leibler (SKL) divergence (Kullback and Leibler, 1951). The pathways for which the prior and posterior distributions are dis-similar are assumed to be impacted more significantly between the two phenotypes. Because of the independence assumption, the distribution of the parameter vector is calculated by multiplying the beta distribution of each of the parameters. The variables of the distributions are calculated using PrimeDB database, or in other words, using the number of journal citations for an interaction type. More details regarding PrimeDB are available in section 2.2. We refer to *beta*(α,β) as the beta distribution with parameters α and β. For the prior distribution, it is assumed that ${\bar{\theta }}_{i}$ ~ *beta*(1, 1), θ_*i*|*j*_ ~ *beta*(*a*_*i*|*j*_, *b*_*i*|*j*_), and ϕ_*i*|*j*_~ *beta*(*b*_*i*|*j*_, *a*_*i*|*j*_), where *a*_*i*|*j*_ and *b*_*i*|*j*_ are the number of journals citing the activation or inhibition between *g*_*i*_ and *g*_*j*_, respectively. For the posterior distribution, it is assumed that ${\bar{\theta }}_{i}$ ~ *beta*(${\bar{n}}_{i}$, *n* − ${\bar{n}}_{i}$), θ_*i*|*j*_ ~ *beta*(*a*_*i*|*j*_ + *n*_*i*|*j*_, *b*_*i*|*j*_ + *n* − *n*_*i*|*j*_), and ϕ_*i*|*j*_ ~ *beta*(*b*_*i*|*j*_ + *n*_*i*|*j*_, *a*_*i*|*j*_ + *n* − *n*_*i*|*j*_), where *n* is the total number of microarray experiments, *n*_*i*_ is the number of experiments in which *g*_*i*_ is up-regulated, and *n*_*i*|*j*_ is the number of experiments in which the pairs *g*_*i*_ and *g*_*j*_ are concordant in up or down regulation. An extension to this method is proposed in which the variables of beta distributions are not calculated by the input as fixed numbers but are assumed to follow exponential distributions. In this case, the parameters of the exponential distributions are estimated from PrimeDB and the input data. It is claimed that this additional probability layer will lead to more robust results. For genes that have more than one parent the majority rule is used to calculate the distribution. The output of this method is the list of pathways scored by the SKL divergence. The lower the score is the more impacted the pathway is assumed to be.

## 4. Output

Although the goal of the pathway analysis should be a ranked list of pathways as a unified output, not all tools reviewed here provide this. Some methods, such as MetPA provide a list of pathways with 2 *p*-values for each pathway, leaving the user to face the task of deciding which *p*-value to trust or how to deal with trade-offs between the two values. Among the methods that rank predefined pathways from public knowledge bases, some methods, such as TopologyGSA, DEgraph, NetGSA, and ACST, find “important” sub-pathways and rank the mixed list of pathways and sub-pathways. In PARADIGM, for each detailed network created by the method based on the input pathway, a matrix of *p*-values is provided as the output. In this matrix, columns are samples and rows are component nodes of the network. Each element of this matrix indicates how likely it is for the node to be in any of the three states comparing the two phenotypes: (1) significantly active, (2) significantly inactive, or (3) have an insignificant change. These scores can be used as substitutes for log fold changes and, as proposed in Vaske et al. (2010), can be input into a non-topology-based gene set analysis algorithm to rank the pathways. Other options to use these scores are either to apply a simple averaging or counting function on the scores of the significant genes to score the pathway, or they can be used to cluster the genes into groups with similar behavior. These clusters of genes can be used to further analyze different features assigned to samples to find group-specific features.

Pathway-Guide offers capability to identify so called “coherent chains of perturbation propagation,” which are to be interpreted as putative mechanisms that are compatible with (and therefore could explain) all measured changes throughout the entire biological system investigated. Even though unique among all other tools and potentially very useful, this capability is completely independent from the pathway ranking provided based on the perturbation and enrichment types of evidence. Therefore, it is possible for pathways that are significant not to contain such coherent signaling cascades, and conversely, pathways that may contain such cascades may not be significant.

In many input data sets, the samples are labeled based on different parameters. The parameters can have qualitative discrete values such as, tumor and normal tissue, or quantitative continuous values such as, survival time of the cell or drug concentration used to treat the tissue. For analysis methods that provide a pathway score for each sample, such as TAPPA and PathOlogist, the pathway activities can be interpreted based on the sample labels. NetGSA (Shojaie and Michailidis, 2010) offers more labeling options in addition to phenotype-based binary labels. The method provides simultaneous tests of multiple hypotheses based on these labels or temporal pathway score correlation to assess the significance of pathways. The rest of the pathway analysis methods compare the pathways using a single qualitative binary label corresponding to the two phenotypes. Methods such as TopoGSA, MetPA, and the hypergeometric extension of CePa calculate one score for each pair of input samples comparing the two phenotypes, while others provide one score for the whole input data set.

Some of the methods provide a graphical display of their results. This is primarily done for the analysis methods which have the ability to provide more than one score for each pathway. For example, analysis methods like TopoGSA have an additional option to compare the properties of the input dataset to predefined datasets corresponding to known functional processes from public databases in a comparative plot. As a result, a summary of network topological properties is displayed for all gene/protein sets in 2D and 3D plots. This functionality allows the user to visually identify an input similar to the original one, based on the plots or based on a tabular ranking using a numerical score to quantify the similarity across all topological properties. Similarly, analysis methods such as Pathway-Guide, Pathway-Express, SPIA, and MetPA which provide two scores (topology based and gene set enrichment) can use a 2D plot to illustrate the distribution of both scores for the analyzed pathways.

From the perspective of the output, the result pathway analysis methods is typically a ranked list of pathways. However, there are tools that only provide nodes scores and further use this scores as input for an existing gene set analysis method. Visual display of the results is a welcomed addition provided by Pathway-Express, Pathway-Guide, SPIA, TopoGSA and MetPA.

## 5. Implementation

The mathematical model presented in Section 3 for each analysis approach is independent of its implementation as a software package. Although the main strength of an approach lies in its algorithm, its implementation can have an important role in reaching the full potential of that approach, as well as in gaining acceptance among the users. Practicality, user-friendliness, output format, and type of interface are all to be considered. Depending on the desired availability and intended audience, a software package may be implemented as standalone or web-based.

Web-based tools run the analyses on a remote server providing computational power and a graphical interface. On the user side, experiment datasets are uploaded, and on the server side, the tool performs the analysis. The results are displayed by the browser in the format provided by the tool. The output of most pathway analysis methods is a ranked list of pathways or sub-pathways. MetPA, THINK-Back-DS and, EnrichNet are among the methods that have web-based implementations. The major advantage of web-based tools is that they are user-friendly and do not require a separate local installation procedure.

Standalone tools need to be installed on local machines which often requires administrative skills. Advantages include instant availability that does not require internet access. Most standalone tools depend on full or partial copies of public pathway databases, stored locally, and need to be updated periodically. Methods like Pathway-Guide, ScorePAGE, SPIA, TAPPA, PathOlogist, NetGSA, TopologyGSA, PWEA, ACST, BPA, and GANPA are in this category. Moreover, there are some methods available both as web-based and standalone, including Pathway-Express, MetaCore, TopoGSA, CePa, and PARADIGM.^5^ Another major advantage of standalone tools are the security and privacy of the experiment data.

The programming language and style used for implementation plays an important role in the acceptance of a method. Software tools that are neatly implemented, packaged, and available online are more appealing compared to those that do not have ready-to-use implementations. Many of the methods surveyed here are implemented in the R programming language and are available as software packages either from bioconductor.org, cran.r-project.org, or the author's website. Their popularity among biologists and bioinformaticians is due to the fact that many bioinformatics dedicated packages are available in R. Pathway-Express (as part of ROntoTools), SPIA, TopoGSA, TopologyGSA, GANPA, DEGraph, NetGSA, ACST, CePa, and ScorePAGE are among those methods. Pathway-Guide, Pathway-Express, TAPPA, and THINK-Back-DS have an implementation in Java, which provides a GUI with self explanatory functionality for users with less software development experience. This allows users to customize the graphical display of the results, using functionalities such as zoom or rotation. CePa has a web-based implementation in Perl in addition to its R standalone package. The MATLAB programming language is used for implementation of methods like PathOlogist, BPA, and NetGSA in order to calculate more complex equations. Other programming languages like C and C++ are also used to implement pathway analysis methods such as PARADIGM and PWEA, which theoretically provide better speed and allow for efficient coding. A summary of the mathematical models and implementation details for the surveyed methods is presented in Table 2.

**Table 2:** **Comparison of topology-based pathway analysis methods using criteria related to the mathematical model and implementation**.

**Method name**	**Graph model**	**Scoring method**	**Availability**	**License**	**Language**	**Available from**
ScorePAGE	Single-type, undirected	Hierarchical, similarity	Standalone	N/A	R	on demand
MetaCore^*^	Single-type, directed	Hierarchical, graph measures	Web-based, Standalone	Thomson Reuters	Java	Reuters, 2013
Pathway-Express	Single-type, directed	Hierarchical, graph measures	Web-based, Standalone	free^**^	Java, R	Drăghici et al., 2013
TAPPA	Single-type, undirected	Hierarchical, NNV	Standalone	N/A	Java	N/A
PathOlogist	Multi-type, directed	Hierarchical, probability	Standalone	CC-BY	MATLAB	Greenblum et al., 2013
Pathway-Guide^*^	Single-type, directed	Hierarchical, graph measures	Standalone	Advaita Corporation, 2013	Java	Advaita Corporation, 2013
SPIA	Single-type, directed	Hierarchical, graph measures	Standalone	GPL (>=2)	R	Tarca et al., 2013
NetGSA	Single-type, directed	Mutivariate, hypothesis test	Standalone	GPL-2	R	Shojaie, 2013
PWEA	Single-type, undirected	Hierarchical, similarity	Standalone	free^**^	C++	Hung, 2013
TopoGSA	Single-type, undirected	Hierarchical, graph measures	Web-based	free^**^	PHP, R	Glaab et al., 2013
PARADIGM	Multi-type, directed	Hierarchical, probability	Web-based, Standalone	free^**^ (standalone) UCSC-CGB (web-based)	C	Vaske and Benz, 2013b, Vaske and Benz, 2013a
TopologyGSA	Single-type, moral undirected	Mutivariate, hypothesis test	Standalone	AGPL-3	R	Massa and Sales, 2013
DEGraph	Single-type, undirected	Mutivariate, hypothesis test	Standalone	GPL-3	R	Jacob et al., 2013
MetPA	Single-type, directed	Hierarchical, graph measures	Web-based	free^**^	PHP, R	Xia, 2013
BPA	Single-type, DAG	Mutivariate, Bayesian network	Standalone	free^**^	MATLAB	Isci, 2013
GANPA	Single-type, undirected	Hierarchical, graph measures	Standalone	GPL-2	R	Fang et al., 2013
BAPA-IGGFD	Single-type, DAG	Mutivariate, Bayesian network	Standalone	N/A	R	N/A
CePa	Single-type, directed	Hierarchical, graph measures	Web-based, Standalone	GPL (>= 2)	R	Gu, 2013b,Gu, 2013a
THINK-Back-DS	Single-type, directed	Hierarchical, graph measures	Web-based, Standalone	free^**^	Java	Farfán et al., 2013b, Farfán et al., 2013a
TBScore	Single-type, directed	Hierarchical, normalized node value (NNV)	N/A	N/A	N/A	N/A
ACST	Single-type, directed	Hierarchical, NNV	Standalone	CC-BY	R	Mieczkowski et al., 2013
EnrichNet	Single-type, undirected	Hierarchical, graph measures	Web-based	free^**^	PHP	Glaab, 2013

* commercial methods;

** free for academic and non-commercial use; UCSC-CGB – the University of California Santa Cruz Cancer Genome Browser;

N/A No publicly available implementation, Graph model indicates whether the graph which is remodeled to be suitable for the scoring method is single-type or multi-type and whether it is directed or undirected. DAG stands for directed acyclic graph. The moral graph is described in Section 3.1. Scoring method encloses the mathematical model used in the analysis to score nodes and graphs. A detailed description is presented in Section 3.2. Implementation indicates the existence of a standalone or web-based implementation of the method. License represents the license under which the software is available. GPL - GNU General Public License, AGPL - GNU Affero General Public License, CC-BY - Creative Commons license. Language represents the programming language used for the implementation. Available from points to the paper or url associated with the given tool.

From the accessibility perspective, web-based tools have the advantage of being available from any location as long as there is an internet connection and a browser available. Also, the update is almost seamless to the client. This makes the user's task easy and enables collaboration. Users all over the world can use the same method without the burden of installing it or keeping it up-to-date. The downside, once the internet connection fails, the tool is unavailable. However, there are methods that provide both web-based and standalone implementations and these are: MetaCore, Pathway-Express, PARADIGM, CePa and THINK-Back-DS.

## 6. Conclusions

Pathway analysis is a core strategy of many basic research, clinical research, and translational medicine programs. Emerging applications range from targeting and modeling disease networks to screening chemical or ligand libraries, to identification/validation of drug target interactions for improved efficacy and safety (Arrell and Terzic, 2010). The integration of molecular interaction information into pathway analysis represents a major advance in the development of mathematical techniques aimed at the evaluation of systems perturbations in biological entities. Out of the 22 topological pathway analysis methods presented here, 15 were published in the last 3 years, evidence that there is great interest and desire for progress in this area.

The important milestones in pathway analysis reflected by this survey are: the first pathway analysis method for metabolic networks (Rahnenführer et al., 2004), the first method for signaling pathway and the first method able to take into consideration the pathway topology (Khatri et al., 2005; Drăghici et al., 2007), the first application of topology-based multivariate hypothesis tests (Shojaie and Michailidis, 2009), and the first analysis able using multi-type graphs from heterogeneous sources (Vaske et al., 2010). In this paper, analysis methods were compared according to types of input, scoring algorithms, results, and user accessibility. Each of these aspects presents its own particular challenges.

The validation of pathway analysis results is an important challenge researchers face when trying to develop such methods. While biologists are needed to verify the pathway analysis results, they depend on pathway analysis methods to support their hypotheses. Most efficient progress will occur with a high level of communication and collaboration between experiment biologists, annotators, pathway designers, bioinformaticians, and computer scientists. As pathway knowledge becomes more complete, the challenge of leveraging this information to extract biological insight from high throughput data will be redefined. Until then, advances will be incremental. Gold standard experiment data sets, designed to affect specific pathways in predefined ways, will be necessary to be able to assess the efficiency of new methods.

Another challenge we mentioned in this survey is that the same biological pathways are represented differently from one pathway database to another. In particular, we pointed out the complications arising from inconsistent conversions for representing interactions among the different pathway databases, and the current efforts to address the problem through the creation of unified formats. However, none of the tools is compatible with all database formats, requiring either modification of pathway input or alteration of the underlying algorithm in order to accommodate the differences. As an example, a study by Vaske and others (Vaske et al., 2010) attempts to compare SPIA (Tarca et al., 2013) with their tool PARADIGM, by re-implementing SPIA in C, and forcing its compatibility with NCI-PID pathways. Grave implementation errors are present in the C version of SPIA, invalidating the comparison. A solution to overcome this challenge could be the development of a unified globally accepted pathway format. Another possible solution is to build conversion software tools that can translate between pathway formats. Some attempts exist to use BIO-PAX as the lingua franca for this domain.

Biological networks are divided in various categories containing complementary information. Signaling and signal transduction are captured by signaling pathways, while biochemical interactions are presented in metabolic pathways. In addition, the protein interaction knowledge bases contain different types of interaction information, complementary to the others. The majority of pathway databases are manually curated and change slowly, but they are evolving toward greater content and accuracy, with new prototype formats being proposed. There is no analysis method that takes advantage of the information stored in all of these different sources. Few of the methods surveyed here use either signaling or metabolic pathways in addition to PPI networks. Promising developments include the integration of multiple component types and interaction types, each with specific properties. Although the information is less reliable, non-curated high-throughput protein interaction data is also proving useful, as protein interaction data can be used to support or filter results.

High-throughput technologies, developed for biological experiments, are improving in accuracy. However, they are still prone to error and the resulting data includes a significant amount of noise. In addition, these technologies produce various types of data among which are genome variations, mRNA level, metabolite concentration, or protein abundance. Each of these data types provides meaningful yet incomplete information regarding specific biological phenomena. The next challenge is to be able to integrate such diverse types of data.

Another challenge is the oversimplification that characterizes many of the models provided by pathway databases. In principle, each type of tissue might have different mechanisms so generic, organism-level pathways present a somewhat simplistic description of the phenomena. Furthermore, signaling and metabolic processes can also be different from one condition to another, or even from one patient to another. Understanding the specific pathways that are impacted in a given phenotype or sub-group of patients should be another goal for the next generation of pathway analysis tools.

Interpreting biological experimental data is also challenging due to inaccurate assumptions. For instance, most current pathway models show cascades of signals or biochemical processes next to one another, in time-agnostic diagrams. In reality, these phenomena happen over time, and often at different time scales. Furthermore, many data sets offer only a snapshot in time, at a particular moment. Almost by definition, such a frozen snapshot cannot properly capture and show the effect of successive events that take place over time.

The graphical scoring methods presented in this paper are representative of the techniques available for future methods. We expect to see greater use of different types of data, in addition to greater use of data mining and machine learning which will lead to more sophisticated topology-based pathway analysis methods.

It is important to (re-)state that the goal of this paper was to survey the main topology-based techniques and methods available to identify the most significant pathways in a comparison between phenotypes. In other words, the goal was to identify, categorize and review these methods *without attempting to assess their performance*. A critical assessment and ranking will be the subject of a later publication. A natural tendency would be to try to use the various criteria used here to compare various methods and thus establish even a partial ordering. For instance, if method X uses only one type of input (e.g., pathways from KEGG) while method Y uses two types of input (e.g., pathways from KEGG as well as PPI data), one might be tempted to conclude that method Y is somewhat more powerful than method X. Similarly, some methods use a subset of DE genes while others use the entire set of measured values. Again, it may be tempting to informally conclude that the later methods are more powerful since, they take more data into consideration or because they eliminate the need for a selection of DE genes. It is our opinion that such inferences and partial orderings are not advisable and should not be attempted based on the information presented in this paper. A proper assessment of these methods should be focused on their ability to identify the pathways that are truly impacted in the given phenotypes, and not based on superficial characteristics or number of features of one type or another.

### Conflict of interest statement

Sorin Drăghici is the founder and CEO of Advaita Corporation, a Wayne State University spin-off that commercializes Pathway-Guide, one of the pathway analysis tools mentioned in this article. The other authors declare that the research was conducted in the absence of any commercial financial relationships that could construed as a potential conflict interest.

## Footnotes

^1^Such experiments are currently offered by a number of academic cores and private companies for a fixed and reasonable fee. Examples of such cores include: Duke Proteomics Core Facility, Johns Hopkins Microarray Core, CMMB Research Facility (Univ. of South Florida), MD Anderson Core Facility, UCLA DNA Microarray Core Facility, Stanford Genomic Resources, Dana-Farber Microarray Core, etc.

^2^BAPA-IGGFD (Zhao et al., 2012) and TBScore (Ibrahim et al., 2012) acronyms were assigned to the respective methods, in this manuscript, for ease of reference.

^3^http://bioconductor.org/packages/release/bioc/html/ROntoTools.html

^4^The moral graph of a DAG is the undirected graph created by adding an (undirected) edge between all parents of the same node (sometimes called marrying), and then replacing all directed edges by undirected edges. The name stems from the fact that, in a moral graph, two nodes that have a common child are required to be married by sharing an edge.

^5^The web-based implementation is only available as part of TCGA while the standalone is available only as C++ source code that needs to be compiled and deployed locally.
